# Supplementary material for: Assessment of Hematologic and Biochemical Parameters for Healthy Commercial Pigs in China
Source: Animals (Basel). 2022 Sep 18;12(18):2464. doi: 10.3390/ani12182464 (PMC9494985; doi:10.3390/ani12182464)
Supplement: Supplementary file 1 [file animals-12-02464-s001.zip › Supplementary Tables_ST1, ST3, ST4.pdf]

Table S1. Descriptive statistics of blood parameters for commercial pigs in China.

Variable	Unit	N	Mean	Sd	Median	Trimmed	Min	Max	Se
WBC	10^9/L	193	16.19	4.05	16.28	16.19	5.58	26.90	0.29
RBC	10^{12}/L	192	6.82	0.69	6.82	6.85	4.93	8.68	0.05
HGB	g/dL	193	113.12	11.66	112.00	112.66	86.00	149.00	0.84
HCT	%	193	38.41	3.99	38.40	38.34	30.20	47.50	0.29
MCV	fL	192	56.57	4.10	56.70	56.60	41.80	72.60	0.29
MCH	pg	194	16.66	1.44	16.40	16.54	12.10	22.40	0.10
MCHC	g/dL	194	294.42	12.24	293.00	294.08	265.00	325.00	0.88
CHCM	g/dL	194	332.13	13.30	333.00	332.40	299.00	373.00	0.95
CH	pg	194	18.75	1.79	18.85	18.73	12.80	25.40	0.13
RDW	%	194	18.99	2.43	18.25	18.58	15.90	29.60	0.17
HDW	g/dL	192	21.00	1.98	20.60	20.84	17.50	32.50	0.14
PLT	10^9/L	193	260.15	138.45	248.00	252.29	29.00	724.00	9.91
MPV	fL	192	9.58	1.42	9.40	9.52	5.60	15.00	0.10
%NEUT	%	192	35.31	9.58	36.15	35.33	11.70	60.90	0.69
%LYMPH	%	192	50.09	10.75	49.95	50.63	6.70	73.80	0.77
%MONO	%	190	6.95	3.13	6.75	6.71	0.60	17.50	0.23
%EOS	%	192	4.53	2.25	4.05	4.23	1.10	16.90	0.16
%BASO	%	193	0.43	0.22	0.40	0.39	0.20	1.50	0.02
%LUC	%	194	1.90	1.08	1.70	1.81	0.30	5.00	0.08
#NEUT	10^9/L	192	5.66	1.96	5.56	5.63	1.11	11.19	0.14
#LYMPH	10^9/L	194	8.10	2.79	8.21	8.11	0.78	14.29	0.20
#MONO	10^9/L	193	1.13	0.59	1.03	1.07	0.15	3.36	0.04
#EOS	10^9/L	191	0.77	0.48	0.65	0.70	0.14	4.15	0.03
#BASO	10^9/L	191	0.07	0.04	0.06	0.06	0.02	0.28	0.01
#LUC	10^9/L	193	0.30	0.17	0.27	0.28	0.04	0.88	0.01
TP	g/dL	193	61.95	12.70	57.70	61.54	42.50	90.70	0.91
ALB	g/dL	193	32.45	5.93	33.40	32.74	19.60	45.30	0.42
AST	U/L	193	41.94	15.21	39.00	39.95	17.00	100.00	1.09
ALT	U/L	193	42.72	10.70	41.00	41.64	23.00	77.00	0.77
ALP	U/L	190	31.02	26.62	26.00	27.19	1.00	158.00	1.92
TC	mg/dL	194	82.44	18.21	81.45	81.64	42.49	179.44	1.30
TG	mg/dL	192	42.32	18.26	39.17	40.83	9.86	104.44	1.31
GLU	mg/dL	193	73.96	24.76	74.00	74.29	16.10	127.10	1.77
CREA	mg/dL	194	98.75	35.85	90.00	93.08	43.00	240.00	2.56
HDL	mg/dL	194	43.54	8.72	42.68	43.13	15.77	76.96	0.62
LDL	mg/dL	194	58.81	17.93	59.01	58.25	22.70	176.00	1.28
BUN	mg/dL	193	5.11	2.68	4.40	4.77	1.60	13.50	0.19
GGT	U/L	194	76.49	45.31	63.50	70.13	17.00	279.00	3.24
CK	U/L	190	888.57	989.36	604.50	688.90	199.00	8986.00	71.40

WBC = white blood cell, RBC = red blood cell, HGB = hemoglobin, HCT = hematocrit, MCV = mean corpuscular volume, MCH = mean corpuscular hemoglobin, MCHC = mean corpuscular hemoglobin concentration, CHCM = cellular hemoglobin concentration mean, CH = corpuscular hemoglobin, RDW = red cell distribution width, HDW = hemoglobin distribution width, PLT = platelets, MPV = mean platelet

volume, NEUT = neutrophil, LYMPH = lymphocyte, MONO = monocytes, EOS = eosinophil, BASO = basophilic granulocyte, LUC = unstained large cells, TP = Total Protein, ALB = Albumin, AST = Aspartate Transaminase, ALT = Alanine Aminotransferase, ALP = Alkaline Phosphatase, TC = Total Cholesterol, TG = Triglyceride, GLU = Glucose, CREA = Creatinine, HDL = High-Density Lipoprotein, LDL = Low Density Lipoprotein, BUN = Blood Urea Nitrogen, GGT = Gamma-Glutamyl Transpeptidase, CK = Creatine Kinase '#' = absolute value, '%' = percentage value. Sd = standard deviation, Se = standard error.

Table S3. Comparison of hematologic parameters between nursery pigs at different ages. Significant differences are indicated by different lowercase letters ($p < 0.05$) and uppercase letters ($p < 0.01$).

Variable	Unit	days			
		20	30	40	50
**WBC**	10^9/L	11.51^{A}±0.76	15.97^{B}±0.6	15.06^{B}±0.6	20.08^{C}±0.62
**RBC**	10^{12}/L	6.86^{A}±0.11	7.17^{B}±0.09	6.38^{C}±0.09	6.61^{AC}±0.09
**HGB**	g/dL	116.64^{A}±2.39	112.10^{AB}±1.86	108.18^{BC}±1.87	105.33^{C}±1.94
**HCT**	%	39.26^{a}±0.81	37.29^{b}±0.63	36.17^{b}±0.63	36.18^{b}±0.66
**MCV**	fL	57.03^{A}±0.81	52.27^{B}±0.62	56.65^{A}±0.62	54.77^{C}±0.65
**MCH**	pg	16.99^{A}±0.26	15.78^{B}±0.2	16.89^{A}±0.21	15.94^{B}±0.21
**MCHC**	g/dL	296.96^{A}±1.81	301.63^{B}±1.41	298.36^{AB}±1.42	291.00^{C}±1.47
**CHCM**	g/dL	326.82±2.28	324.93±1.78	321.71±1.78	321.70±1.85
**CH**	pg	18.68^{A}±0.31	16.98^{B}±0.24	18.13^{AC}±0.24	17.57^{BC}±0.25
**RDW**	%	20.54^{A}±0.49	22.69^{B}±0.38	18.98^{C}±0.38	17.59^{D}±0.4
**HDW**	g/dL	20.74±0.41	21.21±0.32	21.38±0.32	20.85±0.33
**PLT**	10^9/L	236.50^{a}±36.46	289.30^{ab}±28.35	258.22^{b}±28.99	356.61^{a}±29.55
**MPV**	fL	9.34±0.32	9.40±0.25	9.86±0.25	9.51±0.27
**%NEUT**	%	42.49^{A}±2.06	39.87^{AB}±1.62	34.52^{C}±1.6	36.42^{BC}±1.71
**%LYMPH**	%	43.54^{AB}±2.38	46.62^{AB}±1.82	42.20^{A}±1.82	48.71^{B}±1.94
**%MONO**	%	7.50^{A}±0.6	5.62^{B}±0.47	11.82^{C}±0.47	7.75^{A}±0.48
**%EOS**	%	3.70±0.51	4.68±0.39	4.80±0.39	4.69±0.4
**%BASO**	%	0.35±0.03	0.33±0.02	0.40±0.02	0.39±0.02
**%LUC**	%	2.28^{AB}±0.24	1.82^{A}±0.18	2.70^{B}±0.18	1.94^{A}±0.19
**#NEUT**	10^9/L	4.85^{A}±0.42	6.41^{B}±0.33	5.20^{A}±0.32	7.23^{B}±0.34
**#LYMPH**	10^9/L	4.83±0.52	7.33±0.4	6.48±0.4	9.44±0.42
**#MONO**	10^9/L	0.86^{A}±0.12	0.88^{A}±0.09	1.84^{B}±0.09	1.55^{C}±0.1
**#EOS**	10^9/L	0.41^{a}±0.12	0.77^{b}±0.09	0.95^{b}±0.1	0.95^{b}±0.1
**#BASO**	10^9/L	0.04^{A}±0	0.05^{AB}±0	0.06^{B}±0	0.08^{C}±0
**#LUC**	10^9/L	0.27^{a}±0.04	0.30^{a}±0.03	0.42^{b}±0.03	0.36^{ab}±0.03

Table S4. Comparison of biochemical parameters between nursery pigs at different ages. Significant differences are indicated by different lowercase letters ($p < 0.05$) and uppercase letters ($p < 0.01$).

Variable	Unit	days			
		20	30	40	50
**TP**	g/dL	50.18^{A}±0.79	48.17^{B}±0.66	52.54^{C}±0.68	54.49^{D}±0.72
**ALB**	g/dL	32.85^{A}±0.73	29.71^{B}±0.6	26.60^{C}±0.63	24.79^{D}±0.66
**AST**	U/L	60.67^{A}±2.84	45.89^{B}±2.36	44.36^{B}±2.45	40.57^{B}±2.6
**ALT**	U/L	50.24^{A}±2.1	36.33^{B}±1.74	46.88^{AC}±1.81	43.63^{C}±1.92
**ALP**	U/L	60.72^{A}±4.62	59.72^{A}±3.78	40.41^{B}±3.99	29.35^{B}±4.23?

**TC**	mg/dL	100.72^{A}±3.96	60.92^{B}±3.25	80.30^{C}±3.42	81.35^{C}±3.62
**TG**	mg/dL	42.29^{A}±2.67	34.05^{B}±2.19	54.46^{C}±2.34	51.42^{C}±2.44
**GLU**	mg/dL	92.19^{A}±2.85	79.66^{B}±2.37	94.30^{A}±2.46	98.06^{A}±2.61
**CREA**	mg/dL	95.49^{A}±2.49	83.16^{B}±2.04	60.36^{C}±2.15	86.23^{B}±2.28
**HDL**	mg/dL	55.06^{A}±1.76	37.62^{B}±1.44	43.05^{C}±1.52	38.23^{B}±1.61
**LDL**	mg/dL	73.02^{A}±4.14	36.26^{B}±3.4	57.36^{C}±3.58	63.45^{AC}±3.79
**BUN**	mg/dL	3.49^{A}±0.17	3.48^{A}±0.14	2.26^{B}±0.14	3.81^{A}±0.15
**GGT**	U/L	58.15^{A}±8.78	95.89^{B}±7.2	90.00^{B}±7.58	133.30^{C}±8.03
**CK**	U/L	802.50^{A}±93.45	477.10^{B}±74.78	1078.00^{C}±79.9	614.00^{AB}±84.62
